# Caregiver-mediated exercises with e-health support for early supported discharge after stroke (CARE4STROKE): A randomized controlled trial

**DOI:** 10.1371/journal.pone.0214241

**Published:** 2019-04-08

**Authors:** Judith D. M. Vloothuis, Marijn Mulder, Rinske H. M. Nijland, Quirine S. Goedhart, Manin Konijnenbelt, Henry Mulder, Cees M. P. M. Hertogh, Maurits van Tulder, Erwin E. H. van Wegen, Gert Kwakkel

**Affiliations:** 1 Amsterdam Rehabilitation Research Centre, Reade, Amsterdam, The Netherlands; 2 Department of Rehabilitation Medicine, Amsterdam Neuroscience and Amsterdam Movement Sciences, Amsterdam UMC, Vrije Universiteit Amsterdam, Amsterdam, The Netherlands; 3 AptaVivar, Hengelo, The Netherlands; 4 Department of General Practice and Elderly Care Medicine and the EMGO Institute for Health and Care Research, Amsterdam UMC, Vrije Universiteit Amsterdam, Amsterdam, The Netherlands; 5 Department of Health Sciences & Amsterdam Movement Sciences, Faculty of Science, VU University, Amsterdam, The Netherlands; 6 Department of Physiotherapy & Occupational Therapy, Aarhus University Hospital, Aarhus, Denmark; 7 Department of Physical Therapy and Human Movement Sciences, Northwestern University, Evanston, IL, United States of America; University of Glasgow, UNITED KINGDOM

## Abstract

**Background and purpose:**

We designed an 8-week caregiver-mediated exercise program with e-health support after stroke (CARE4STROKE) in addition to usual care with the aim to improve functional outcome and to facilitate early supported discharge by increasing the intensity of task specific training.

**Methods:**

An observer-blinded randomized controlled trial in which 66 stroke patient-caregiver couples were included during inpatient rehabilitation. Patients allocated to the CARE4STROKE program trained an additional amount of 150 minutes a week with a caregiver and were compared to a control group that received usual care alone. Primary outcomes: self-reported mobility domain of the Stroke Impact Scale 3.0 (SIS) and length of stay (LOS). Secondary outcomes: motor impairment, strength, walking ability, balance, mobility and (Extended) Activities of Daily Living of patients, caregiver strain of caregivers, and mood, self-efficacy, fatigue and quality of life of both patients and caregivers. Outcomes were assessed at baseline, 8 and 12 weeks after randomization.

**Results:**

No significant between-group differences were found regarding SIS-mobility after 8 (β 6.21, SD 5.16; *P* = 0.229) and 12 weeks (β 0.14, SD 2.87; *P* = 0.961), and LOS (*P* = 0.818). Significant effects in favor of the intervention group were found for patient’s anxiety (β 2.01, SD 0.88; *P* = 0.023) and caregiver’s depression (β 2.33, SD 0.77; *P* = 0.003) post intervention. Decreased anxiety in patients remained significant at the 12-week follow-up (β 1.01, SD 0.40; *P* = 0.009).

**Conclusions:**

This proof-of concept trial did not find significant effects on both primary outcomes mobility and LOS as well as the secondary functional outcomes. Treatment contrast in terms of total exercise time may have been insufficient to achieve these effects. However, caregiver-mediated exercises showed a favorable impact on secondary outcome measures of mood for both patient and caregiver.

**Clinical trial registration:**

NTR4300, URL– http://www.trialregister.nl/trialreg/admin/rctview.asp?TC=4300.

## Introduction

Stroke rehabilitation aims to reduce long-term dependency and to allow patients to return to the community. [1] Meta-analyses have shown that increased intensity of training leads to better functional outcome in stroke patients. [2, 3] However, resources for rehabilitation services after stroke (mostly staff) are becoming increasingly scarce and it proves to be difficult to offer a sufficient dose of exercise therapy. [4] Therefore, alternative treatment strategies are needed to increase the amount of exercise therapy without increasing healthcare costs. [5, 6] Caregiver-mediated exercises, in which stroke patients perform exercises with a caregiver, may be a promising approach. In addition, caregiver-mediated exercises have the potential to facilitate early supported discharge (ESD) [7–9] by smoothing the transition from inpatient care to the home setting and providing opportunities to continue exercise therapy in the community. Since independence in transfers and/or gait is an important factor in enabling discharge to the community [10], focus of caregiver-mediated exercises on patients’ independence in terms of regaining mobility and gait-related activities is useful.

A recent Cochrane review, in which 333 patient-caregiver couples were included for meta-analysis, found very low to moderate quality evidence in favor of caregiver-mediated exercises for standing balance, walking distance, and quality of life. However, the included nine studies were heterogeneous in terms of quality, methodology, content, timing and duration of the intervention, warranting further investigation. [11] A recent phase IV trial in 14 hospitals in India failed to show positive effects of a family-led rehabilitation program on the modified Rankin Scale (mRS) when compared to usual care. In this program, rehabilitation professionals were educated to train nominated family members. The nominated family member practiced upper limb function, mood management, positioning, transfers and mobility with the patient. [12] This broad-spectrum program may have been too diluted and too weak, and the dose of augmented exercise therapy insufficient, to introduce significant shifts in mRS scores. In addition, no strict procedure for caregiver selection was described, and the number of caregiver training sessions seems too small to provide progressive and high-quality exercise training for the patient. [13]

To increase adherence and self-efficacy of the patient-caregiver couple, and to facilitate remote coaching and monitoring by the rehabilitation team, [14] the present proof-of-concept trial supported caregiver-mediated exercises with e-health methods and combined it with tele-rehabilitation services. [15]

We hypothesized that the CARE4STROKE program would lead to better self-reported mobility, with a clinically important difference of 5 points on the mobility domain of the Stroke Impact Scale (SIS, version 3.0) [16] and a reduced length of stay (LOS) for stroke patients compared to usual care, without increasing caregiver burden. In addition, we hypothesized that psychosocial functioning and mobility related functional outcomes, such as balance and lower limb function, would significantly improve by the CARE4STROKE program.

## Methods

### Design

The CARE4STROKE trial was an observer-blinded multicenter randomized controlled trial in which a caregiver-mediated exercises program with e-health support, combined with tele-rehabilitation, in addition to usual care, was compared with a control group that received usual care alone. Participants were recruited from hospital stroke units, rehabilitation centers and nursing homes in the Netherlands. Design, inclusion and exclusion criteria, outcome measures and data analysis have been described in detail elsewhere and are summarized here. [15] Methods and results are reported in accordance with the CONSORT statements. [17]

Patients were randomly allocated (1:1) to either the intervention or the control group. An online randomization procedure, using a computerized minimization algorithm with ‘type of setting’ as only covariate, was applied by an independent researcher who was not involved in the treatment program. Subsequently, the independent researcher informed the treating physical therapists about the treatment allocation of the patient (and caregiver). The allocation schedule was only visible for the coordinating researchers who were not involved in inclusion or assessment of participants.

**Fig 1:**
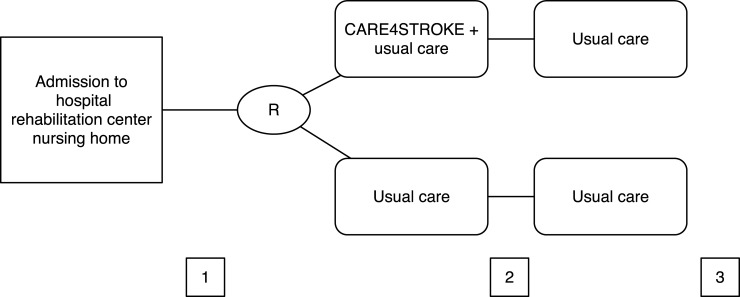
Study design. R = Randomization. 1 = Measurement 1, baseline, before start of the intervention. 2 = Measurement 2, end of intervention (8 weeks post randomization). 3 = Measurement 3, follow up (12 weeks post randomization).

All assessments were performed at baseline and 8 and 12 weeks post randomization by 2 observers (MM and QG), who were trained in standardized outcome assessment. Observers were blinded for treatment allocation. (Fig 1). Participants and physical therapists could not be masked for group allocation.

The study protocol was approved by the Medical Ethics Review Committee of the Slotervaart Hospital and Reade (number NL34618.048.12) and was registered in the Dutch trial register as NTR4300, registered 2 December 2013 (http://www.trialregister.nl/trialreg/admin/rctview.asp?TC=4300). All participants provided written informed consent. There were no changes in trial design [15] during the study period, except the removal of the Caregiver Strain Index as an exclusion criterion (> 4 points), since caregiver-mediated exercises might actually reduce caregiver strain and it would therefore be unfortunate to deny caregivers to participate in the intervention.

### Participants and setting

Patients were recruited in the participating hospitals (N = 4), rehabilitation centers (N = 2) and geriatric rehabilitation departments of nursing homes (N = 7). All patients admitted were screened by participating physiotherapists and physicians. When patient and caregiver seemed to be eligible, they received a participant information letter explaining the study and the consequences of participating. During a subsequent session with one of the research assistants (MM and QG), the research assistant checked the following in- and exclusion criteria and obtained informed consent.

Patients were eligible if they (1) had a stroke according the WHO definition [18]; (2) had lived independently before the stroke; (3) were planned to be discharged home; (4) were able to follow instructions (MMSE score > 18 points) (5) had a Functional Ambulation Score (FAC) < 5 and (6) were willing and able to appoint a caregiver who wanted to participate in the program (with a maximum of two caregivers). A caregiver was defined as someone close to the patient who was willing and able to do exercises together with the patient, for example a partner, family member or friend. This caregiver was not a professional and was not paid for his/her efforts. Patients were asked to appoint one or two preferred caregivers, thereafter inclusion criteria for the caregivers were checked. These inclusion criteria for the caregiver were: (1) being medically stable and (2) being physically able to perform the exercises together with the patient. Inclusion criteria for both patients and caregivers were (1) aged 18 years or older; (2) written informed consent; (3) ability to understand Dutch or English (at a sufficient level to understand instructions); (4) sufficiently motivated to participate in the caregiver-mediated exercise program; and (5) a score of <11 on the ‘depression’ domain of the Hospital Anxiety and Depression Scale (HADS).

An exclusion criterion for both patients and caregivers was a serious comorbidity that interfered with mobility training, for example a severe cardiopulmonary illness or a disabling orthopedic comorbidity of the lower extremity. To finally determine the suitability of patients and caregivers, an intake exercise session with a trained physical therapist was scheduled prior to inclusion. During this session the therapist judged if the patient-caregiver couple was able to exercise adequately and safely together. A short checklist, evaluating these criteria, was used by the physical therapist. (S2 Text_checklist physiotherapist intake exercise session)

### Intervention

The content of the CARE4STROKE program is reported in accordance with the TIDieR guidelines [19, 20] and has been published elsewhere in more detail. [21] Briefly, the program consisted of 8 weeks of exercise therapy, executed with a caregiver, in addition to usual care following the current guidelines in the Netherlands. [2] The exercise program was composed by a trained physical therapist during weekly sessions. The therapist could choose from 37 standardized exercises aimed at improving mobility, presented in an e-health application (‘app’).

For each patient, exercises were combined into a patient-tailored, progressive training regimen, related to the patient goals. Patient-caregiver couples were encouraged to contact the coordinating therapist using tele-rehabilitation services like telephone, video conferencing or email when appropriate in between the weekly exercise sessions. The patients and their caregivers were instructed to perform the selected set of exercises at least five times a week for 30 minutes. This meant that patients received 20 hours of caregiver-mediated exercises in addition to usual care during the 8-week intervention period. When the patient’s discharge date fell before the anticipated end date of the CARE4STROKE intervention, the program was continued at home. All physical therapists were thoroughly trained in a training course, prior to delivering the CARE4STROKE program.

The participants in the control group received usual care according to the guidelines for physical therapy for patients with stroke of the Royal Dutch Society for Physical Therapy (KNGF). [2] Therapy sessions are designed according to patient goals. Therefore, there were no restrictions with respect to content, time or duration of the physical therapy. Task and context specificity are important aspects of physical therapy after stroke. With that, in current guidelines, exercises are recommended to improve functional outcomes such as standing balance, physical condition, and walking competence.

### Outcome measures

*Primary outcome measures* were the mobility domain of the SIS 3.0 [22, 23] and LOS. LOS was defined as the time from stroke onset to the moment of discharge from the rehabilitation facility.

*Secondary outcome measures* were all other domains of the SIS; Fugl-Meyer motor score of the lower extremity; Motricity Index of the lower extremity leg; Six-minute walking test; Ten-meter walking test; Timed Up and Go test; Berg Balance Scale; Rivermead Mobility Index; Barthel Index; Nottingham Extended ADL scale and mRS, for the patient. Secondary outcome measures for caregivers included the Caregiver Strain Index and Carer Quality of Life Scale. The HADS, Fatigue Severity Scale and General Self-Efficacy Scale were included for both patients and caregivers. In addition, patients and caregivers kept a diary recording exercise times and relevant cost data (e.g. visits to specialists, missed work time). An economic evaluation carried out alongside the randomized controlled trial will be reported on in a separate publication. During the trial, we excluded the personal opinion questionnaire for empowerment from the outcome measures, to reduce the time load of the assessments. Since evidence suggests that adding the five positively phrased items in the Expanded Caregiver Strain Index does not improve the psychometric properties of the Caregiver Strain Index [24], we decided to report the Caregiver Strain Index.

### Statistical analysis

Sample size calculation showed that 66 patients were needed to achieve sufficient statistical power (80%) to detect a significant difference with a two-tailed alpha level of *P*<0.05. [15] We powered the study for a significant reduction of five points (11%) on the SIS mobility domain measured post intervention, with an estimated standard deviation for this population at a maximum of 14 points. [25]

We tested the successful blinding of the assessors for treatment allocation by comparing assessors’ guesses with actual treatment assignment, using a Cohen’s κ statistic.

Data were analyzed according to the intention-to-treat principle and the statistician was kept blinded for group allocation. Missing items were imputed using serial means. Missing values were not imputed if entire questionnaires or scales were missing.

Between-group differences at baseline were studied using Mann-Whitney U tests. Subsequently, main outcomes were compared between the intervention and control groups at 8 and 12 weeks after randomization, using a Generalized Estimating Equations (GEE) model with an exchangeable covariance structure. Time, group, baseline value of the dependent variable, covariates that showed significant differences at baseline and the interaction between group and time were included in the regression model. We calculated β-values and standard errors for the group × time interaction effects and applied a Wald statistic to obtain corresponding *P*-values. All hypotheses were tested two-sided, with an α < 0.05. To test if the model was appropriate, we repeated the analysis with other covariance structures. Differences in LOS were analyzed using a Mann-Whitney U test.

## Results

**Fig 2:**
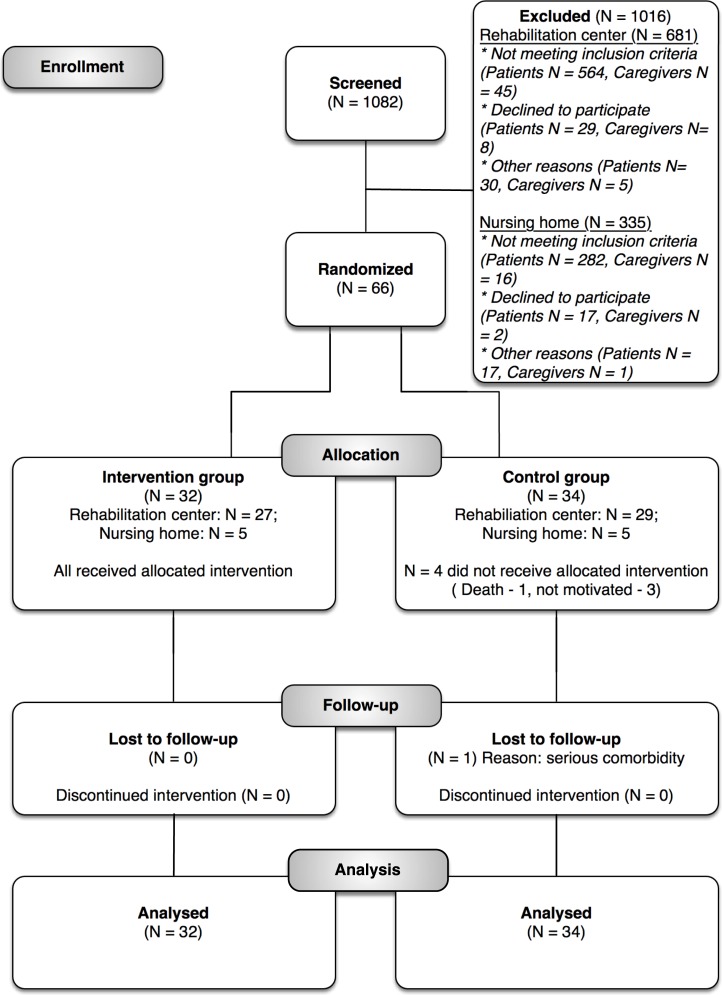
Consort flow diagram.

After screening 1082 patients admitted on the neurological wards of the participating centers, we recruited 66 participants between April 2014 and July 2016. Most patients were excluded because they did not suffer a stroke. (Fig 2) Follow-up measurements were completed in October 2016. Recruitment of patients and numbers of dropouts are presented in the flow chart (Fig 2). Fifty-six of the 66 patients were recruited from rehabilitation centers, whereas 10 patients were recruited from nursing homes and no patients were recruited from participating hospitals. As a result, we did not carry out separate analyses for type of participating center. We found a Cohen’s κ coefficient of 0.3 when comparing observers’ guesses about treatment allocation and actual allocation.

Baseline characteristics of the participants are presented in Table 1. Mean age of the included patients with stroke was 59.9 years (Standard deviation (SD) 14.8). Median time after stroke was 37 days (Interquartile range (IQR) 28–56). There was a significant difference in favor of the control group at baseline regarding SIS communication, and a significant baseline difference in favor of the control group regarding depression (HADS) of the caregivers. Both factors were used as covariates in the main regression analysis.

**Table 1:** Baseline characteristics of participants.

	Control (N = 34)	Intervention (N = 32)
***Patient***		
Sex, Female / Male	14 / 20	11 / 21
Mean age (SD), years	59.26 (15.01)	60.53 (14.82)
Education, low / high ^1^ ^mv^	20 / 13	21 / 10
Living arrangement, alone / together	10 / 24	10 / 22
Working before stroke, yes / no ^mv^	21 / 12 ^1^ ^mv^	19 / 13
Time after stroke onset in days, median (IQR)	37 (26–55)	36 (28–57)
Stroke type, hemorrhagic / ischemic / SAH	4 / 28 / 2	10 / 22 / 0
Side of stroke, Right / Left / Brainstem	21 / 12 / 1	16 / 16 / 0
Recurrent stroke, yes / no	3 / 31	2 / 30
Aphasia, yes / no	6 / 28	8 / 24
Hemianopia, yes / no	5 / 29	5 / 27
Visual spatial neglect, yes / no ^mv^	9 / 23	10 / 22
MMSE (0–30), median (IQR)	28 (25–29)	27 (24–29)
FAC (0–5), median (IQR)	1.5 (0–3)	2 (0–3)
SIS mobility (0–100), mean (SD)	41.42 (20.45)	49.91 (24.17)
SIS communication (0–100), mean (SD)	87.92 (18.11)	78.57 (23.11)*
***Caregiver***		
Sex, Female / Male	21 / 13	23 / 9
Mean age (SD), years	54.00 (12.26)	53.91 (14.90)
Education, low / high ^1^ ^mv^	13 / 18	14 / 18
Relation to the patient, N (%)		
***Partner***	19 (55.9)	20 (62.5)
***Child***	7 (20.6)	7 (21.9)
***Friend***	1 (2.9)	1 (3.1)
***Parent***	2 (5.9)	1 (3.1)
***Sibling***	4 (11.8)	2 (6.3)
***Volunteer***	1 (2.9)	-
***Other family member***	-	1 (3.1)
Currently working, yes / no ^mv^	19 / 12	23 / 9
HADS depression (0–21), mean (SD)	2.88 (2.54)	4.28 (2.99)*
HADS anxiety (0–21), mean (SD)	4.44 (3.40)	5.68 (2.99)
CSI (0–13), mean (SD)	4.53 (2.11)	5.42 (2.66)

SD: standard deviation, IQR: interquartile range, mv: missing values, IQR: interquartile range, SAH: subarachnoid hemorrhage, MMSE: mini mental state examination, FAC: functional ambulation categories, SIS: Stroke Impact Scale, HADS: hospital anxiety and depression scale, CSI: Caregiver Strain Index

^1^ education low: none/primary school/secondary school/ intermediate vocational

education

education high: higher vocational education, college, university

* P < 0.05

Patients in the intervention group reported a median of 1190 minutes of additional exercise therapy with a caregiver (*P* = 0.002). However, when the total amount of self-reported exercise time was calculated (i.e. time during therapy + independent + with a nurse + with a caregiver), there was no significant difference between the intervention and control groups (median 4060 minutes versus 3735 minutes; *P* = 0.098). S1 Table in the supporting information shows that these findings did not change when using different imputation methods.

Absolute values, Beta (SE) scores and *P*-values for the time x group interaction effect after 8 and 12 weeks are presented in S2 Table in the supporting information. No significant time x group interaction effect was found for the primary outcome measure of SIS mobility at week 8 (β 6.21, SD 5.16; *P* = 0.229) or week 12 (β 0.14, SD 2.87; *P* = 0.961), nor was a significant difference found in LOS (*P* = 0.818). Patients in the control group were admitted to inpatient stay for a mean of 117 (SD 54) days, versus a mean of 117 (SD 50) days for the intervention group. Significant interaction effects in favor of the intervention group were found regarding mood, viz. for HADS anxiety of the patient after 8 weeks (β 2.01, SD 0.88; *P* = 0.023) and 12 weeks (β 1.01, SD 0.40; *P* = 0.009), and for HADS depression of the caregiver after 8 weeks (β 2.33, SD 0.77; *P* = 0.003). No significant interaction effects were found for any of the other secondary outcome measures. Findings did not differ when using a GEE model with a different covariance structure. No adverse events were reported. Underlying data is available in the supporting information (S3 Table CARE4STROKE database).

## Discussion

In this observer-blinded randomized proof-of-concept trial comparing a caregiver-mediated exercises program with e-health support combined with tele-rehabilitation (CARE4STROKE) to usual care alone, we found no differential effect with respect to the primary outcome measures of self-perceived mobility (SIS-mobility) and LOS. In addition, we did find that the CARE4STROKE intervention was feasible and safe.

Insufficient treatment contrast in terms of total exercise time might explain the lack of effects found on functional outcome measures. However, a significant difference in favor of the intervention group was observed, in terms of decreased patient anxiety and caregiver depression. These significant treatment effects might be explained by the significant difference in exercise time with a caregiver. In contrast to exercise therapy supported by health professionals or exercising alone, practicing together with a partner, family member or friend seems to have a positive effect on psychosocial functioning of both patients and caregivers. The incidence of anxiety in stroke patients [26] and depression in their caregivers [27] is significantly higher than in healthy age-matched controls. In addition, depressive as well as anxiety symptoms are predictors of lower quality of life of patients [28, 29], and of long-term burden and emotional problems of caregivers. [30] So, interventions that target anxiety and depression symptoms are important. The observed HADS values in our participants were in the low range (lower values correspond to less depression or anxiety), which might be caused by our inclusion criteria of <11 points on the HADS depression subscale. However, the found effects of caregiver-mediated exercises on the HADS values exceed minimal clinically important differences [31] and are therefore worth further exploring. Future trials may even consider including patients and caregivers who are mildly depressed or anxious, because caregiver-mediated exercises might help to decrease these symptoms. Of course, only with very strict monitoring during the caregiver-mediated exercises program.

These positive effects of CARE4STROKE on mood are also in line with previously reported positive effects of caregiver-mediated exercises on caregiver strain [32] and the quality of life of patients. [11] The present findings are also in line with a trial using the same protocol and running parallel in Adelaide, Australia (N = 63). [33] They found a significant reduction of caregiver fatigue and improved self-efficacy in the caregiver-mediated exercises group. In a qualitative study using semi-structured interviews we performed alongside the CARE4STROKE trial, participants reported that caregiver-mediated exercises made them feel more actively involved in the rehabilitation process, and prepared them for the home situation. [34] This might, at least in part, explain the reduced anxiety and depression we found in the present trial and so caregiver-mediated exercises may smooth the transition from the rehabilitation center to the home situation, which patients and caregivers report as a significant hurdle. [35, 36]

In this trial we did not find an effect on LOS and thus on facilitation of ESD. However, in view of the impact on mood, we argue that caregiver-mediated exercises might be an important component in future more protocolized ESD programs. First, to prepare patients and caregivers for discharge to their own home situation. Second, to continue exercising at home. The latter could probably well be supported by e-health tools and tele-rehabilitation services. It would be interesting to further expand this and study its effects.

The CARE4STROKE intervention has now been studied in two different parts of the world (i.e. Australia and Western Europe). In addition, caregiver-mediated exercises interventions have been studied in countries like India [12] and Ireland [32]. All these countries have quite different (socio-geographical) circumstances and health care systems. Future studies should investigate cross-cultural differences with respect to effectiveness of caregiver-mediated exercise programs in different health care systems. [13]

This study has several limitations. First, our hypothesis is based on 1200 minutes of additional exercise time by the patient-caregiver couples. Although the intervention group approached the intended dose of caregiver-mediated exercises (1190 minutes), there was no significant difference in the total amount of exercise time between the intervention and control group. Patients in the control group reported more exercise time with a therapist or nurse and also performed exercises with a caregiver. Therefore, there might have been insufficient treatment contrast to improve mobility and other functional outcomes. This type of contamination is often seen in stroke rehabilitation trials that require a long recruitment period of several years to finalize. [37, 38]

Second, our sample size calculation was based on the assumption of a standard deviation of 14 points for the SIS mobility. [25] In the current study the standard deviation was approximately 20 points. Our study may therefore be under powered.

Third, although independent mobility is an important factor in enabling discharge to the community, length of inpatient stay is also determined by other, including non-clinical, factors.[39] Interesting however is, that while we did not find differences in LOS, the parallel Australian trial found a 9-day reduction of LOS in a per-protocol analysis of 20 patients who received tele-rehabilitation at home.[33]

Finally, our patients were not included at fixed times after stroke, resulting in variable timings after stroke onset [40], which increased the likelihood of not finding between-group differences. [41–43]

Future full- scale trials should focus on gaining a better understanding of the effects of caregiver-mediated exercises on psychosocial outcome measures and their value for ESD. Outcome measures might be aimed at constructs such as depression, anxiety, empowerment, quality of life and smoothness of transfer to the home situation. Sample size should be larger and to prevent contamination a cluster randomized trial is recommended. [44, 45] In order to advance precision one might consider a repeated measurement design with a longer follow up period. Finally, inclusion and assessments should preferably be done at fixed times post-stroke. [41]

## Conclusions

This proof-of-concept randomized controlled trial showed that the CARE4STROKE program is a feasible approach to exercise with a caregiver. Although no significant differences were found on self-perceived mobility, LOS and functional outcomes, which may be caused by insufficient treatment contrast, CARE4STROKE did have a favorable impact on secondary outcome measures of mood for both patients and caregivers.

## Supporting information

S1 Table Self-reported exercise time over 8 weeks (in minutes), reported as medians and interquartile ranges (IQR). (DOCX)

S2 Table Absolute values of outcomes, reported as means and standard deviations (SD), Beta (SE = Standard error) and *P*-values of outcome measures. (DOCX)

S3 Table CARE4STROKE database. (XLSX)

S1 Text Consort checklist. (DOC)

S2 Text Checklist physiotherapist intake exercise session. (DOCX)

S3 Text Study protocol CARE4STROKE. (PDF)
