# Supplementary material for: Caregiver-mediated exercises with e-health support for early supported discharge after stroke (CARE4STROKE): A randomized controlled trial
Source: PLoS One. 2019 Apr 8;14(4):e0214241. doi: 10.1371/journal.pone.0214241 (PMC6453481; doi:10.1371/journal.pone.0214241)
Supplement: S1 Table — (DOCX) [file pone.0214241.s001.docx]

|  | **Without imputation** | | | **With imputation^1^** | | | **With imputation^2^** | | |
| --- | --- | --- | --- | --- | --- | --- | --- | --- | --- |
|  | ***Control (N=25^mv^)*** | ***Intervention (N=25^mv^)*** | ***P value*** | ***Control (N=28^mv^)*** | ***Intervention (N=30^mv^)*** | ***P value*** | ***Control (N=29^mv^)*** | ***Intervention (N=31^mv^)*** | ***P value*** |
| **During therapy** | 2152.5  (1300.0 -2846.25) | 2010.0  (1395.0 – 2920.0) | 0.793 | 2295.0  (1340.00 - 2842.50) | 2040.0  (1430.0 – 2940.0) | 0.767 | 2152.5  (1252.5 – 2838.8) | 2010  (1394.0 – 2920.0) | 0.688 |
| **Independent** | 570.0  (275.0 – 475.0) | 400.0  (105.0 – 1292.5) | 0.462 | 560.0  (272.5 – 1002.5) | 510.0  (105.0 – 1200.0) | 0.607 | 550.0  (275.0 – 985.0) | 550.0  (110.0 -1200.0) | 0.773 |
| **With nurse** | 130.0  (0 – 475.0) | 20.0  (0 -120.0) | 0.119 | 85.0  (0.0 – 420.0) | 20.0  (0.0 – 142.5) | 0.200 | 80.0  (0.0 - 380.0) | 20.0  (0.0 – 140.0) | 0.145 |
| **With caregiver(s)** | 350.0  (95.0 – 1065.0) | 1150.0  (850.0 – 1500.0) | 0.004^*^ | 595.0  (152.5 – 1117.5) | 1195.0  (885.0 – 1533.3) | 0.002^*^ | 480.0  (115.0 – 1105.0) | 1190.0  (870.0 – 1530.0) | 0.002^*^ |
| **Total** | 3860.0  (3153.5 – 4522.5) | 4060.0  (3472.5 – 5275) | 0.237 | 3742.5  (3184.0 – 4477.5) | 4150.0  (3498.75 – 4915.0) | 0.141 | 3735.0  (3153.5 – 4475.0) | 4060.0  (3525.0 – 4850.0) | 0.098 |

* *P* < 0.05

mv: missing values

^1^ Missing items were only imputed when a minimum of 5 weeks of the diary had been filled out

^2^ All missing items were imputed, except when the entire diary had not been filled out
