# Supplementary material for: Caregiver-mediated exercises with e-health support for early supported discharge after stroke (CARE4STROKE): A randomized controlled trial
Source: PLoS One. 2019 Apr 8;14(4):e0214241. doi: 10.1371/journal.pone.0214241 (PMC6453481; doi:10.1371/journal.pone.0214241)
Supplement: S2 Text — (DOCX) [file pone.0214241.s005.docx]

**Checklist physiotherapist intake exercise session**

- Is the caregiver able to sufficiently support the patient (physically and mentally)?
- Are the exercises performed safely together by patient and caregiver?
- Is the collaboration going well between patient and caregiver?
- Is the patient sufficiently instructable?
- Do both patient and caregiver know what is expected from them regarding the exercises?
- Do both patient and caregiver know what is expected from them regarding the diary?
