# Supplementary material for: Caregiver-mediated exercises with e-health support for early supported discharge after stroke (CARE4STROKE): A randomized controlled trial
Source: PLoS One. 2019 Apr 8;14(4):e0214241. doi: 10.1371/journal.pone.0214241 (PMC6453481; doi:10.1371/journal.pone.0214241)
Supplement: S3 Text — (PDF) [file pone.0214241.s006.pdf]

RESEARCH PROTOCOL

Care4Stroke program: Caregiver mediated exercises with e-health support for early supported discharge after stroke.

Care4Stroke program: Caregiver mediated exercises with e-health support for early supported discharge after stroke

**Protocol ID**	**NL34618.048.12**
**Short title**	**CARE4STROKE**
**Version**	**9**
**Date**	**06-11-2014**
**Coordinating investigator/project leader**	Prof. dr. G.Kwakkel<br>VU medisch centrum, afdeling revalidatiegeneeskunde<br>Postbus 7057<br>1007MB Amsterdam<br>Email: g.kwakkel@vumc.nl
**Principal investigator**	**Reade**<br>Drs JDM Vloothuis, MD<br>Physiatrist, department acquired brain injury<br>Email: j.vloothuis@reade.nl<br><br>**VU Medical Centre**<br>Prof dr G.Kwakkel<br>Email: g.kwakkel@vumc.nl
**Sponsor**	Mrs M Schinkel<br>Manager divisie revalidatie volwassenen<br>Reade<br>Overtoom 283<br>1054 HW, Amsterdam<br><br>Prof.dr.G.Kwakkel<br>Hoogleraar neurorevalidatie<br>Afdeling revalidatiegeneeskunde VUMC<br>De Boelelaan 1117<br>1081 HV Amsterdam<br><br>M.R. Wisse MBA<br>Directeur Vivium Zorggroep<br>Postbus 6085<br>100 HB Bussum

	Dhr H Mulder<br>Directeur Evocare BV<br>Marijkestraat 7<br>7555 VX Hengelo
**Subsidising party**	ZonMw<br>Programma Doelmatigheidsonderoek<br>Postbus 93245<br>2509 AE Den Haag<br>Contactpersoon: Karen van Liere- Visser<br>Tel: 070-3495272<br>Doelmatigheidsonderzoek@zonmw.nl
**Independent physician**	**Reade:**<br>Christof Smit, MD, Msc<br>Physiatrist<br>Reade<br>Overtoom 283<br>1054 HW, Amsterdam<br>Email: c.smit@reade.nl<br><br>**VUMC:**<br>Dr. Carel Meskers, MD, PhD<br>Physiatrist<br>VUMC, afdeling revalidatiegeneeskunde<br>Postbus 7057<br>1007 MB Amsterdam<br>Email: c.meskers@vumc.nl<br><br>**VIVIUM Naarderheem:**<br>Aafke de Groot<br>Amersfoortsestraatweg 1<br>1411 HB Naarden<br>Tel: 035-6954411

Verpleeghuis het Zonnehuis
Judith Simone Duifs
Specialist ouderengeneeskunde
Zonnehuisgroep Amstelland
Groenelaan 9
1186 AA Amstelveen
tel: 020 - 426 5200

Ziekenhuis Amstelland
Afdeling revalidatiegeneeskunde (valt onder Reade)
Christof Smit, MD, Msc
Revalidatiearts
Reade
Overtoom 283
1054 HW, Amsterdam
Email: c.smit@reade.nl

Onze Lieve Vrouwen Gasthuis
Afdeling revalidatiegeneeskunde (valt onder Reade)
Christof Smit, MD, Msc
Revalidatiearts
Reade
Overtoom 283
1054 HW, Amsterdam
Email: c.smit@reade.nl

Revant revalidatie
Evelien Picard
Revalidatiearts
Brabantlaan 1
4817 JW Breda
Telefoon 076 5797900

Sint Lucas Andreas Ziekenhuis
E.J. Wouda
Neuroloog
Sint Lucas Andreas Ziekenhuis
Jan Tooropstraat 164
1061 AE Amsterdam

Cordaan, locatie Berkenstede en verpleeghuis Slotervaart.
M- Kiebert-Welteroth
Specialist ouderengeneeskunde
Cordaan
De Ruyterkade 7
1013 AA Amsterdam

Evean
T. de Graas
Specialist ouderengeneeskunde
Oostergouw
Koningin Julianaweg 10
1502 DZ Zaandam

PROTOCOL SIGNATURE SHEET

Name	Signature	Date
**Sponsor/ head op department:**<br>***Mrs M. Schinkel, manager Reade***		
**Project leader**<br>***Prof.dr.G.Kwakkel***		

TABLE OF CONTENTS

1. INTRODUCTION AND RATIONALE 11
2. OBJECTIVES 13
3. STUDY DESIGN 14
4. STUDY POPULATION 15
   4.1 Population (base) 15
   4.2 Exclusion criteria 16
   4.3 Sample size calculation 16
5. TREATMENT OF SUBJECTS 17
   5.1 Investigational treatment 17
   5.2 Control treatment 17
   5.3 Use of co intervention 17
6. METHODS 18
   6.1 Study parameters/endpoints 18
      6.1.1 Main study parameters 18
      6.1.2 Other study parameters 19
   6.2 Randomisation, blinding and treatment allocation 19
   6.3 Study procedures 20
   6.4 Withdrawal of individual subjects 20
   6.5 Replacement of individual subjects after withdrawal 20
   6.6 Follow-up of subjects withdrawn from treatment 21
   6.7 Premature termination of the study 21
7. SAFETY REPORTING 22
   7.1 Section 10 WMO event 22
   7.2 Adverse and serious adverse events 22
   7.3 Follow-up of adverse events 23
8. STATISTICAL ANALYSIS 24
   8.1 Univariate analysis 24
   8.2 Multivariate analysis 24
9. ETHICAL CONSIDERATIONS 25
   9.1 Regulation statement 25
   9.2 Recruitment and consent 25
   9.3 Benefits and risks assessment, group relatedness 25
   9.4 Compensation for injury 26
   9.5 Incentives (if applicable) 26
10. ADMINISTRATIVE ASPECTS AND PUBLICATION 27
   10.1 Handling and storage of data and documents 27
   10.2 Amendements 27
   10.3 Annual progress report 27
   10.4 End of study report 27
   10.5 Public disclosure and publication policy 27
11. REFERENCES 28

LIST OF ABBREVIATIONS AND RELEVANT DEFINITIONS

BBS	Berg Balance Scale
BI	Barthel Index
CME	Caregiver mediated exercise
CBO	Centraal begeleidings orgaan
CSI	Caregiver strain index
ESO	European Stroke Organisation
ESD	Early Supported Discharge
FAC	Functional Ambulation Categories
FAME	Family mediated exercises
HADS	Hospital anxiety and depression scale
HSU	Hospital Stroke Unit
LOS	Length of Stay
METC	Medical research ethics committee (MREC); in Dutch: medisch ethische toetsing commissie (METC)
MMSE	Mini Mental State Evaluation
NEADL	Nottingham Extended ADL index
NH	Nursing Home
PT	Physical Therapist
RC	Rehabilitation Centre
RCT	Randomized controlled trial
RMI	Rivermead Mobility Index
(S)AE	(Serious) Adverse Event
SIS	Stroke impact scale
Sponsor	The sponsor is the party that commissions the organisation or performance of the research, for example a pharmaceutical company, academic hospital, scientific organisation or investigator. A party that provides funding for a study but does not commission it is not regarded as the sponsor, but referred to as a subsidising party.
WHO	World Health Organisation
WMO	Medical Research Involving Human Subjects Act (in Dutch: Wet Medisch-wetenschappelijk Onderzoek met Mensen)

SUMMARY

Rationale:

Several systematic reviews have indicated that additional exercise therapy and repetitive task training have a significant effect on functional outcome after stroke. Guidelines therefore conclude that patients in a rehabilitation setting should have the opportunity to get an increase of intensity of therapy. At this moment resources in rehabilitation facilities are not sufficient to meet these recommendations. A new method could be to involve caregivers (partner, family, friends) in exercise training. Previous studies suggest that this form of exercises done with a caregiver can lead to a better functional outcome for the patient and less strain for the caregiver. A critical part will be safety, adherence of the patient and caregiver and continuing support, for which innovative e-health and structured tele-rehabilitation services could be used.

In addition, a recent meta-analysis has shown that early supported discharge with additional services in the community is beneficial for optimizing the transition from the rehabilitation setting to the home situation and is cost-effective by reducing the length of stay of inpatient services, acknowledging that inpatient rehabilitation accounted for about 44% of all care of stroke costs.

Objective: The primary aim of this study is to evaluate the feasibility, clinical effectiveness and cost effectiveness of a caregiver mediated exercises programme combined with e-health services (CARE4STROKE) to improve self-reported health status and reduce the length of stay and costs by allowing early supported discharge of stroke patients to their own home setting.

Study design: randomized controlled trial (RCT).

Study population: 66 stroke patients and their caregivers, admitted in several rehabilitation centers, Hospital Stroke Units and nursing homes in the Netherlands will participate in this study.

Intervention: Participants will be randomly allocated to either 8 weeks of the CARE4STROKE programme in addition to usual care or to 8 weeks of usual care.

Main study parameters/endpoints:

Primary measurements of outcome: 1) Length of Stay and 2) self-reported health-status with the Stroke Impact Scale (SIS version 3.0). Secondary outcomes for included stroke patients are EuroQol-5D (EQ-5D), the Barthel Index, Rivermead Mobility Index, Berg Balance Scale, 5 meter walking speed, 6 minute walking test, Timed Up and Go Test, the Motricity Index (leg), The Fugl-Meyer assessment (leg), Nottingham Extended Activities of Daily Living and modified Rankin Scale. In order to track the daily activity, patients will wear a wireless activity monitor on the wrist one week before and after the intervention. In addition, patients keep a

diary to record adherence to the exercise program and emerging complications. Caregiver burden will be evaluated with the Caregiver Strain Index and Carer Quality of Life Index. For patients and caregivers the Hospital Anxiety and Depression Scale, Fatigue Severity Scale, General self-efficacy scale and Personal Opinion Questionnaire for empowerment will be used. In addition each couple will use a cost diary.

Nature and extent of the burden and risks associated with participation, benefit and group relatedness: Participants in the intervention group get a surplus of 150 minutes of exercise training a week; Caregivers will be involved and will need to allocate time to the programme as well. Care is taken to assure safe performance of the exercises. This will be accomplished by e-health and tele-rehabilitation services and safety instructions and close guidance and coaching of a therapist. The control group will have no additional benefit or risks. Assessments will take place at baseline, after the intervention and at twelve weeks follow up. They consist of questionnaires and tests, taking approximately two hours per assessment.

1. INTRODUCTION AND RATIONALE

The annual rate for first ever stroke in the Netherlands is estimated about 41.000 persons, with a prevalence of about 216.500. [1, 2] The costs incurred by the care of stroke patients are extremely high and are estimated to exceed one billion Euros in the Netherlands. [3] A recent analysis of initial and secondary care costs of Stroke in the United Kingdom showed that inpatient rehabilitation accounted for about 44% of all costs. [4] In addition, a recent meta-analysis involving 14 trials (1957 patients) has shown that early supported discharge (ESD) with additional services in the community is beneficial in optimizing the transition from the rehabilitation setting to independent living and cost-effective by reducing the length of stay (LOS) of inpatient services and long term dependency of stroke patients.[5] Several RCT's [6] and longitudinal studies [7] have shown that ESD is enabled as soon as patients are independent in their transfers and gait, suggesting that ESD is heavily dependent on balance and motor control of the lower limb. Exercise therapy typically focuses on restoring and/or improving motor function and performance of activities of daily living with the aim to enhance independence. Several systematic reviews have indicated that additional exercise therapy and repetitive task training have a significant effect on functional outcome after stroke, concluding that that more exercise therapy is better. [8-13]

Guidelines in the Netherlands conclude therefore that patients who are in a rehabilitation setting should have the opportunity to receive a minimal dose of 40 to 60 minutes exercise therapy.[14] In the same vein, the UK guidelines in the United Kingdom recommends a daily dose of 45 minutes of exercise therapy in the early stages after stroke. [15-17]

At this moment, most patients admitted to hospital stroke units (HSU), rehabilitations Centres (RC) and nursing homes (NH) spend most of their waking time during workdays and weekends being physically inactive or involved in activities that contribute little to their recovery.[18-20] A recent survey in the Netherlands of 91 hospital Stroke Units showed that patients receive about 24 minutes of exercise therapy each working day, whereas a policy for rehabilitation in the weekends is lacking.[21] Acknowledging that the resources (mostly staff) in the HSUs, RCs and NH's are not sufficient to meet the minimal dose of exercise therapy "novel" methods to increase the duration and intensity of exercise therapy with minimal use of resources are needed.[22] For example, we recently showed that task-oriented group training sessions of 6 to 8 stroke patients simultaneously (ie, circuit class training; CCT) given by a physiotherapist and a sport instructor is an equally effective strategy as an individual face-to-face approach for patients suffering from a stroke. [23]

An alternative method could be to involve caregivers in exercise training. The concept of caregiver mediated exercise (CME) has been tested in a pilotstudy in Reade. Given the experience gained in this pilot study, CME can be considered feasible and safe for both patients en caregivers. To date, a limited number of studies [24-28] about CME in patients with

stroke has been published. Three of these are randomized controlled trials. [29-31] Galvin et al [32] studied the added value of a 'family mediated exercise intervention' (FAME) in a HSU. They found that a program of additional daily exercises with a partner aimed at the lower extremity significantly improved the functional outcome of the lower extremity, balance, walking ability and activities of daily living, compared to usual care in a hospital stroke unit. Markedly, the experienced strain of the caregiver measured on the caregiver strain index significantly decreased. One other relevant economic study by Patel et al found that training of the caregiver in the techniques of nursing and facilitation of personal care significantly reduces total health and social care costs for stroke patients over one year, mainly due to reductions in length of hospital stay. [33]
Recently, Gregory et al. recommended a crucial role of e-Health and ICT support for the coordination of caregiver-mediated interventions.[34] However, none of the existing studies however have combined e-Health services with Caregiver Mediated Exercise to enhance Early Supported Discharge. .
The present type I cost-effectiveness study will serve as basis for a future cluster stepped-wedge randomized phase III clinical trial to investigate the cost-effectiveness of the CARE4STROKE program, in which Caregiver Mediated Exercise is combined with e-Health services aimed at Early Supported Discharge
We hypothesize that the CARE4STROKE program will:
1) be feasible and safe to execute;
2) allows ESD of patient to their own home setting with a significant reduced inpatient stay of 1 day for HSU and 7 days for RC/NH.
3) increase patients self-reported health status with concomitant reduced levels of caregiver burden and
4) enhance feelings of empowerment as perceived by patient and partner when compared to usual care..

2. OBJECTIVES

The CARE4STROKE program is directed at improving self-reported health status and reduce LOS and costs by allowing ESD of stroke patients to their own home setting. ESD from setting of admission will be enhanced by: 1) starting CME applied by a PT focused on gait and gait-related activities immediately after admission in a HSU, RC NH and 2) continuous weekly support by a PT in patients own home setting after discharge with support of e-Health services. The e-Health services will contain the following package: 1) an app with CME for 8 weeks, starting immediately after admission; 2) telerehabilitation services allowing instructions of the PT including monitoring progress of patients and adherence of caregiver and 3) telephone and video conferencing based counseling and weekly visits by a coordinating PT from the setting of discharge focused on increasing feelings of empowerment and self-management of the caregiver.

The main aim of the present pilot study is to investigate feasibility, clinical effectiveness and cost-effectiveness of a caregiver-mediated exercise program (CARE4STROKE) combined with e-Health services, to be evaluated in terms of self-reported health status and mean LOS in stroke patients who are admitted at a HSU, RC or NH and discharged to their own home setting, as compared to usual care.

3. STUDY DESIGN

This pilot study has a randomized controlled trial design in a HSU, RC and NH.
Within each center 22 patients will be randomly allocated to either usual care + CARE4STROKE program or usual care. Patients will start immediately after admission, continue for 8 weeks irrespective of time of discharge and will be followed-up for 12 weeks.

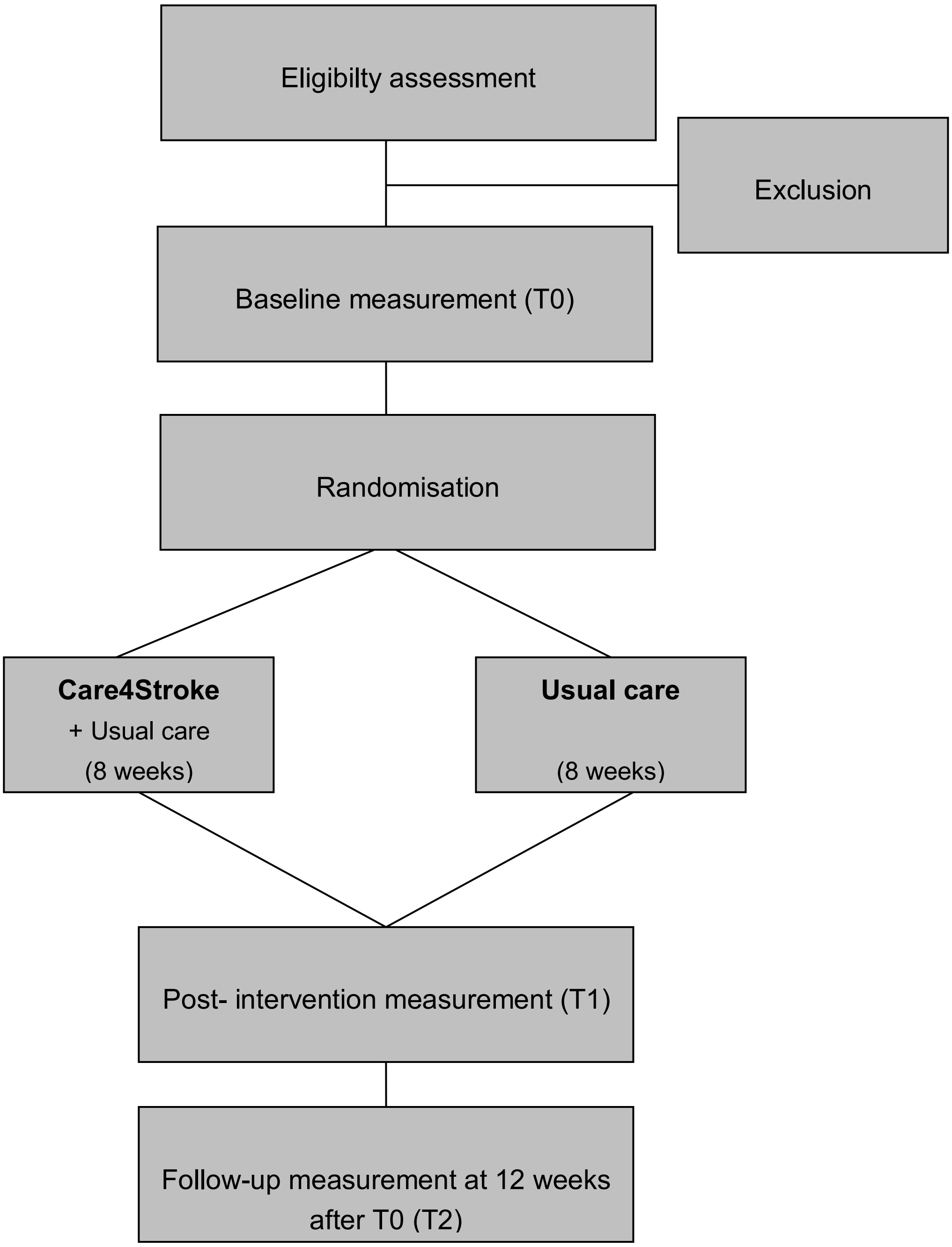

4. STUDY POPULATION

4.1 Population (base)

Sixty-six patients with stroke and their caregiver will be recruited for this study. Patients with stroke, who are admitted to a participating HSU, RC or NH, and their caregivers will be asked to participate in this study at the moment of admission. There will be no restrictions in relation between patient and caregiver, i.e. the caregiver can be a partner, family member, neighbour or other person close to the patient. The patient has to grant explicit permission as to the choice of the caregiver. There will be a maximum of two caregivers per patient.

Inclusion criteria for the patient:

1) 18 years or older
2) written informed consent
3) able to understand the Dutch language (on sufficient level to understand instructions and complete the questionnaires)
4) knowing and able to appoint a caregiver who he/she wants to participate in the programme (with a maximum of two caregivers)
5) living independently before the stroke
6) planned to be discharged home
7) being able to follow instructions (a MMSE score > 18 points)
8) Functional Ambulation Score (FAC) < 5
9) a score of <11 on the domain “depression” of the Hospital Anxiety and Depression Scale (HADS)
10) Motivated for CME

Inclusion criteria for the caregiver:

1) 18 years or older
2) written informed consent
3) able to understand the Dutch language (on sufficient level to understand instructions and complete the questionnaires)
4) sufficiently motivated for CME
5) a score of <11 on the domain “depression” of the Hospital Anxiety and Depression Scale (HADS)
6) medically stable and physically able to perform the exercises together with the patient.
7) No significant Caregiver Strain (<4 SCI)

4.2 Exclusion criteria

Exclusion criteria for both patient and caregiver will be serious comorbidity which interferes with participation.

To determine suitability of both patient and partner, an intake exercise session together with a trained therapist will be scheduled prior to inclusion. The therapist will check the inclusion/exclusion criteria and judge if the exercises can be done adequately and safely.

4.3 Sample size calculation

We expect a significant reduction of 5 points (11%) on the SIS mobility domain [23] [mean 79,4, sd 14] in favor of the experimental training group. We expect that minimally 30 patients are required per arm of the trial. Including 10% dropouts, we suppose that a minimum of 66 stroke patients, (i.e. 22 per centre), are needed to achieve a sufficient statistical power of 80% using a significance level alpha of $p<0.05$.

5. TREATMENT OF SUBJECTS

5.1 Investigational treatment

The CARE4STROKE program consists of eight weeks of complementary exercise therapy done with a caregiver, next to the usual therapy. 31 standardized exercises are available, that can be customized per patient and caregiver into an individualized program. These exercises were devised in collaboration with movement scientists and physical therapists and were shown to be feasible in the pilot study in Reade. The exercises are presented in an E-health application (app) for a tablet. Regular reminders to exercise can be given by the app. The exercises are aimed at improving skills related to walking ability like sitting, standing and making transfers, or are supporting exercises to improve mobility, strength and balance. The patient and their caregiver are asked to do the exercises minimally 5 times a week for 30 minutes on at least both weekend days or the equivalent dosage with an adopted schedule. When the intervention is correctly performed patients will have a surplus of 150 minutes of caregiver mediated therapy a week.
Patients and their caregiver will have a weekly session with a trained therapist. In this session, the participating couple will be instructed as to which exercises should be performed safely during the next week and evaluate the exercises done last week. All patients and caregivers will be supported by a handbook with instructions.
The program starts when the patient is admitted in one of the participating centres. When the discharge date of the patient is earlier than the finishing of the program, the program continues at home with continuous monitoring from the treating therapist.

5.2 Control treatment

The participants in the control group will receive usual care according to the Dutch guidelines for patients with stroke and the Royal Dutch Guidelines of Physical Therapy.

5.3 Use of co intervention

All patients that receive usual care will not be enrolled in other studies. During the study, usual care will be recorded. Participants in both the intervention and control group will use a diary to register every therapy and (self) training session.

6. METHODS

6.1 Study parameters/endpoints

6.1.1 Main study parameters

For the patient:

1) Length of Stay in the HSU, RC or NH, defined as the moment of admittance-moment of discharge. (primary outcome)
2) Self reported health status with the Stroke Impact Scale (SIS version 3.0) [35-38]
3) Quality of life, measured with the EQ-5D. [39]
4) Mobility, measured by the Rivermead mobility index (RMI) [40-42]
5) Independence in performing basic activities of daily living, measured by the Barthel index (BI) [43]
6) Walking ability, assessed with the 5 metre walking speed, the 6 minute walking test and the timed up and go test (TUG). [44, 45]
7) Extended Activities of daily living, measured by the Nottingham extended ADL (NEADL). [46]
8) Functional outcome, measured by Modified Rankin Scale (MRS) dichotomised to good outcome (0-2) or poor outcome (3-6). [47]
9) Balance, assessed with the Berg Balance Scale (BBS) [48, 49]
10) Selectivity lower extremity, assessed with the Fugl Meyer (FM) lower extremity.[50]
11) Strength of the lower limb, assessed with the Motricity Index (MI). [51]
12) The amount of daily activity, assessed with a comfortable and wireless activity monitor on the wrist.

For the caregiver:

13) the experienced strain of the caregiver measured by the Expanded Caregiver strain index. (CSI +) [52-54]
14) Quality of life, measured with the CareQOL.[55, 56]

For both:

15) Amount of (additional) practice done by the couples in the intervention and controlgroup, this will be measured with a diary (see appendix) Problems and adverse events like falls will also be recorded in the diary
16) Personal Opinion Questionnaire for empowerment .

17) Emotional functioning, measured with the Hospital Anxiety and Depression Scale (HADS). [57, 58]
18) Fatigue, measured by the fatigue severity scale. [59]
19) Self-efficacy, measured by the general self-efficacy scale. [60, 61]
20) A cost diary, comprising questions on items such as consultation with neurologists, family doctors, paramedics, re-admission to hospitals or rehabilitation centres, drug use, home care and non-professional support.

Outcome measures will be measured at baseline prior randomization, after the eight week intervention period and again after 12 weeks (follow up) by a blinded assessor who is not involved in training. Length of stay in the rehabilitation center will be reported at discharge of the patient. Self reports in the (cost)diary will take place during the intervention.
To asses the long term costs, some patient will be asked to keep the cost diary until the end of the study in 2016. These data will be obtained by the blinded assessor with telephone interviews.

In addition, at the end of the intervention, semi-structured interviews will be conducted with some patients and caregivers to collect qualitative data regarding the experience of CME.

6.1.2 Other study parameters

Patient and caregiver characteristics will be recorded. Of the patient we will document: age, type of stroke, date of stroke, hemiplegic side, sensory deficits (yes/no), hemianopia (yes/ no) and neglect (yes/no). Of the caregiver we will record: age, relation to patient, work (yes/no), duration of partnership and existing morbidities

6.2 Randomisation, blinding and treatment allocation

In this study patients will be at random to the control or the experimental group using stratified block randomisation. Patients allocated to the experimental group will receive the CARE4Stroke program. The randomisation procedure will be executed by an independent researcher. The study will be registered in the Dutch trial register.

6.3 Study procedures

Every patient with stroke, admitted in one of the participating centres, will be screened for eligibility by their own treating medical specialist to evaluate if participation in this study is possible. On a registration form will be noted: if a person is asked, if this person wants to participate, and what the considerations were. An information letter is handed to patient and caregiver and a reflection period of one week will follow. Eligible patients and their chosen caregiver will be informed in an interview with the primary researcher and a therapist will judge if the caregiver can adequately assist the patient during exercises. Upon inclusion, informed consent will be signed. Thereafter the baseline measurements will be done (T0). The couples will be randomly allocated to either the intervention or control group.
For the couples allocated to the intervention group the intervention starts directly. After eight weeks the same measurements as at baseline will be repeated (T1) for every couple. Follow up measurements will be performed 12 weeks post randomisation.
(see figure 1)
The assessments consist of clinical tests and questionnaires (see outcome measures). The assessments and inclusion will be done by an assessor who is blinded to treatment allocation.
Prior to starting the inclusion of patients, all therapists will be extensively trained in applying the CARE4STROKE programme to optimize standardization with regard to coaching the participants and performing the weekly training sessions in which patient and partner learn the exercises.

6.4 Withdrawal of individual subjects

Participants can leave the study at any time for any reason if they wish to do so without any consequences. The investigator can decide to withdraw a participant from the study for urgent medical reasons. In the patient and caregiver information letter participants will be informed about their right to withdraw from the study or intervention without any explanation

6.5 Replacement of individual subjects after withdrawal

Individual participants will not be replaced after withdrawal.

6.6 Follow-up of subjects withdrawn from treatment

If participants decide to terminate the assigned intervention premature, they will be asked if they want to continue completing the outcome measurements. They can refuse this request without any consequences.

6.7 Premature termination of the study

There are no specific criteria for premature termination of the study.

7. SAFETY REPORTING

7.1 Section 10 WMO event

In accordance to section 10, subsection 1, of the WMO, the investigator will inform the subjects and the reviewing accredited METC if anything occurs, on the basis of which it appears that the disadvantages of participation may be significantly greater than was foreseen in the research proposal. The study will be suspended pending further review by the accredited METC, except insofar as suspension would jeopardise the subjects' health. The investigator will take care that all subjects are kept informed.

7.2 Adverse and serious adverse events

Adverse events are defined as any undesirable experience occurring to a subject during the study, whether or not considered related to the experimental treatment. All adverse events reported spontaneously by the subject or observed by the investigator or his staff will be recorded.

A serious adverse event is any untoward medical occurrence or effect that at any dose:

- results in death;
- is life threatening (at the time of the event);
- requires hospitalisation or prolongation of existing inpatients' hospitalisation;
- results in persistent or significant disability or incapacity;
- is a new event of the trial likely to affect the safety of the subjects, such as an unexpected outcome of an adverse reaction, lack of efficacy of an IMP used for the treatment of a life threatening disease, major safety finding from a newly completed animal study, etc.

All SAEs will be reported through the web portal *ToetsingOnline* to the accredited METC that approved the protocol, within 15 days after the sponsor has first knowledge of the serious adverse reactions.

SAEs that result in death or are life threatening should be reported expedited. The expedited reporting will occur not later than 7 days after the responsible investigator has first knowledge of the adverse reaction. This is for a preliminary report with another 8 days for completion of the report.

7.3 Follow-up of adverse events

All adverse events will be followed until they have abated, or until a stable situation has been reached. Depending on the event, follow up may require additional tests or medical procedures as indicated, and/or referral to the general physician or a medical specialist.

8. STATISTICAL ANALYSIS

.

8.1 Univariate analysis

Baseline characteristics as described in par 6.1.2 will be presented and between group differences will be studied to determine whether groups are comparable at baseline.
When these data are not normally distributed, non parametric wilcoxon signed rank sum test will be used. When the data are normally distributed student t-test will be used. The α will be set at 0,05.

8.2 Multivariate analysis

The main outcomes as described in par 6.1.1 will be compared between the intervention and control group at the different timepoints using multilevel regression analysis. Time, group, location and possible significant baseline values will be added to the model.

8.3 Cost-effectiveness analysis

A cost effectiveness and a cost-utility analysis will be performed The primary outcome of the trial, length of Stay, in combination with the information of the cost diary will be used in the cost effectiveness analysis. Unit costs will be taken from national sources.

In the costs utility-analysis the outcome measure is quality-adjusted life-years (QALYs) based on the Dutch tariff for the Euro(Qol). [39]

9. ETHICAL CONSIDERATIONS

9.1 Regulation statement

The study will be conducted according to the principles of the Declaration of Helsinki 59th WMA General Assembly (Seoul, October 2008) and in accordance with the Medical Research involving Human Subjects Act (WMO).

9.2 Recruitment and consent

In the first week of admittance in one of the participating centres, every patient with stroke will be screened by their own treating medical specialist to evaluate if participation in this study is possible. Eligible patients and their chosen caregiver will be informed in an interview with the primary researcher and a therapist will judge if the caregiver can adequately assist the patient during exercises. A reflection period will follow. Upon inclusion, a second interview with the primary researcher will follow and informed consent will be signed. Next the baseline measurements will be done. Randomisation will follow and treatment starts as soon as possible after that.

9.3 Benefits and risks assessment, group relatedness

When the CARE4STROKE programme is followed patients will have a surplus of 150 minutes of training a week. Literature shows this to be beneficial for functional outcome after stroke. Next to that caregivers will be involved and will most likely feel in control because they can help. This may lower their experienced strain.
The exercise programme can be tailor made for the patient and caregiver by the therapist. Given the experience gained in the pilot study, the intervention can be considered as safe for both patients and caregivers. The small risk of adverse events will be minimized since a therapist will judge if the caregiver can adequately assist the patient during exercises before inclusion. In addition, safety instructions are provided in the app and there is a close guidance of a therapist during the intervention period. Also instructions are described what to do in case of an adverse event. The control group will have no additional benefit or risks. Assessments will take place at baseline, after the intervention and at twelve weeks post randomisation. They consist of questionnaires and tests, taking approximately two hours per assessment.

9.4 Compensation for injury

The sponsor/investigator has a liability insurance which is in accordance with article 7, subsection 6 of the WMO.

Each participating centre has an insurance which is in accordance with the legal requirements in the Netherlands (Article 7 WMO and the Measure regarding Compulsory Insurance for Clinical Research in Humans of 23th June 2003). This insurance provides cover for damage to research subjects through injury or death caused by the study.

1. € 450.000,-- (i.e. four hundred and fifty thousand Euro) for death or injury for each subject who participates in the Research;
2. € 3.500.000,-- (i.e. three million five hundred thousand Euro) for death or injury for all subjects who participate in the Research;
3. € 5.000.000,-- (i.e. five million Euro) for the total damage incurred by the organisation for all damage disclosed by scientific research for the Sponsor as 'verrichter' in the meaning of said Act in each year of insurance coverage.

The insurance applies to the damage that becomes apparent during the study or within 4 years after the end of the study.

9.5 Incentives (if applicable)

Subjects will not receive special incentives or additional treatments next to the assigned intervention during their participation in this study. Any travel costs incurrent for participation will be reimbursed.

10. ADMINISTRATIVE ASPECTS AND PUBLICATION

10.1 Handling and storage of data and documents

All research data will be stored in Reade, center for rehabilitation and rheumatology. The data will be kept 15 years. All research files will have a code, which makes the file anonymously. Only the research team is able to trace the data to the individual subjects by using a subject identification code list. The outcome measurements can also be reported in the medical file of the patient when he/ she gives his/ her consent.

10.2 Amendements

Amendments are changes made to the research after a favourable opinion by the accredited METC has been given. All amendments will be notified to the METC that gave a favourable opinion.

10.3 Annual progress report

The sponsor/investigator will submit a summary of the progress of the trial to the accredited METC once a year. Information will be provided on the date of inclusion of the first subject, numbers of subjects included and numbers of subjects that have completed the trial, serious adverse events/ serious adverse reactions, other problems, and amendments.

10.4 End of study report

The investigator will notify the accredited METC of the end of the study within a period of 8 weeks. The end of the study is defined as the last patient's last visit.
In case the study is ended prematurely, the investigator will notify the accredited METC, including the reasons for the premature termination.
Within one year after the end of the study, the investigator/sponsor will submit a final study report with the results of the study, including any publications/abstracts of the study, to the accredited METC.

10.5 Public disclosure and publication policy

All the publications of this study will be public.

11. REFERENCES

Reference List

(1) Franke CL, Vaartjes I, Bots ML. Volksgezondheid Toekomst Verkenning. Nationaal Kompas Volksgezondheid; 2011.

(2) Vaartjes I, Dis van I, Visseren FLJ, Bots ML. Hart en Vaatziekten in Nederland 2010- Cijfers over leefstijl en risicofactoren, ziekte en sterfte. Den Haag: Nederlandse Hartstichting; 2010.

(3) Slobbe LCJ, Smit JM, Groen J, Poos MJJC, Krommer GJ. Kosten van ziekten in Nederland 2007: Trends in de Nederlandse zorguitgaven 1999-2010. Bilthoven: RIVM; 2013.

(4) Luengo-Fernandez R, Gray AM, Rothwell PM. A population-based study of hospital care costs during 5 years after transient ischemic attack and stroke. *Stroke* 2012 December;43(12):3343-51.

(5) Fearon P, Langhorne P. Services for reducing duration of hospital care for acute stroke patients. *Cochrane Database Syst Rev* 2012;9:CD000443.

(6) Langhorne P, Bernhardt J, Kwakkel G. Stroke rehabilitation. *Lancet* 2011 May 14;377(9778):1693-702.

(7) Kollen B, van dP, I, Lindeman E, Twisk J, Kwakkel G. Predicting improvement in gait after stroke: a longitudinal prospective study. *Stroke* 2005 December;36(12):2676-80.

(8) French B, Thomas L, Leathley M, Sutton C, McAdam J, Forster A, Langhorne P, Price C, Walker A, Watkins C. Does repetitive task training improve functional activity after stroke? A Cochrane systematic review and meta-analysis. *J Rehabil Med* 2010 January;42(1):9-14.

(9) Galvin R, Murphy B, Cusack T, Stokes E. The impact of increased duration of exercise therapy on functional recovery following stroke--what is the evidence? *Top Stroke Rehabil* 2008 July;15(4):365-77.

(10) Kwakkel G, van PR, Wagenaar RC, Wood DS, Richards C, Ashburn A, Miller K, Lincoln N, Partridge C, Wellwood I, Langhorne P. Effects of augmented exercise therapy time after stroke: a meta-analysis. *Stroke* 2004 November;35(11):2529-39.

(11) Kwakkel G. Impact of intensity of practice after stroke: issues for consideration. *Disabil Rehabil* 2006 July 15;28(13-14):823-30.

(12) Langhorne P, Bernhardt J, Kwakkel G. Stroke rehabilitation. *Lancet* 2011 May 14;377(9778):1693-702.

(13) Veerbeek JM, Koolstra M, Ket JC, van Wegen EE, Kwakkel G. Effects of augmented exercise therapy on outcome of gait and gait-related activities in the first 6 months after stroke: a meta-analysis. *Stroke* 2011 November;42(11):3311-5.

(14) Richtlijn diagnostiek, behandeling en zorg voor patienten met een beroerte. Kwaliteitsinstituut voor de gezondheidszorg; 2008.

(15) Intercollegiate Stroke Working Party. National clinical guideline for stroke, 3rd edition. London: Royal College of Physicians; 2008.

(16) Guidelines for Management of Ischaemic Stroke and Transient Ischaemic Attack 2008 The European Stroke Organization (ESO). Executive Committee and the ESO Writing Committee; 2008.

(17) SIGN guideline 118, management of patients with stroke: rehabilitation, prevention and management of complications, and discharge planning. 2010.

(18) Bernhardt J, Dewey H, Thrift A, Donnan G. Inactive and alone: physical activity within the first 14 days of acute stroke unit care. *Stroke* 2004 April;35(4):1005-9.

(19) Smith P, Galea M, Woodward M, Said C, Dorevitch M. Physical activity by elderly patients undergoing inpatient rehabilitation is low: an observational study. *Aust J Physiother* 2008;54(3):209-13.

(20) West T, Bernhardt J. Physical activity in hospitalised stroke patients. *Stroke Res Treat* 2012;2012:813765.

(21) Otterman NM, van der Wees PJ, Bernhardt J, Kwakkel G. Physical therapists' guideline adherence on early mobilization and intensity of practice at dutch acute stroke units: a country-wide survey. *Stroke* 2012 September;43(9):2395-401.

(22) Langhorne P, Bernhardt J, Kwakkel G. Stroke rehabilitation. *Lancet* 2011 May 14;377(9778):1693-702.

(23) Port van de I, Wevers LE, Lindeman E, Kwakkel G. Effects of circuit training as alternative to usual physiotherapy after stroke: randomised controlled trial. *BMJ* 2012;344:e2672.

(24) Forster A, Young J, Nixon J, Kalra L, Smithard D, Patel A, Knapp M, Monaghan J, Breen R, Anwar S, Farrin A. A cluster randomized controlled trial of a structured training programme for caregivers of inpatients after stroke (TRACS). *Int J Stroke* 2012 January;7(1):94-9.

(25) Galvin R, Cusack T, O'Grady E, Murphy TB, Stokes E. Family-mediated exercise intervention (FAME): evaluation of a novel form of exercise delivery after stroke. *Stroke* 2011 March;42(3):681-6.

(26) Kalra L, Evans A, Perez I, Melbourn A, Patel A, Knapp M, Donaldson N. Training carers of stroke patients: randomised controlled trial. *BMJ* 2004 May 8;328(7448):1099.

(27) Maeshima S, Ueyoshi A, Osawa A, Ishida K, Kunimoto K, Shimamoto Y, Matsumoto T, Yoshida M. Mobility and muscle strength contralateral to hemiplegia from stroke: benefit from self-training with family support. *Am J Phys Med Rehabil* 2003 June;82(6):456-62.

(28) Osawa A, Maeshima S. Family participation can improve unilateral spatial neglect in patients with acute right hemispheric stroke. *Eur Neurol* 2010;63(3):170-5.

(29) Forster A, Young J, Nixon J, Kalra L, Smithard D, Patel A, Knapp M, Monaghan J, Breen R, Anwar S, Farrin A. A cluster randomized controlled trial of a structured training programme for caregivers of inpatients after stroke (TRACS). *Int J Stroke* 2012 January;7(1):94-9.

(30) Galvin R, Cusack T, O'Grady E, Murphy TB, Stokes E. Family-mediated exercise intervention (FAME): evaluation of a novel form of exercise delivery after stroke. *Stroke* 2011 March;42(3):681-6.

(31) Kalra L, Evans A, Perez I, Melbourn A, Patel A, Knapp M, Donaldson N. Training carers of stroke patients: randomised controlled trial. *BMJ* 2004 May 8;328(7448):1099.

(32) Galvin R, Cusack T, O'Grady E, Murphy TB, Stokes E. Family-mediated exercise intervention (FAME): evaluation of a novel form of exercise delivery after stroke. *Stroke* 2011 March;42(3):681-6.

(33) Patel A, Knapp M, Evans A, Perez I, Kalra L. Training care givers of stroke patients: economic evaluation. *BMJ* 2004 May 8;328(7448):1102.

(34) Gregory P, Alexander J, Satinsky J. Clinical telerehabilitation: Application for physiatrists. Physical Medicine and Rehabilitation 3, 647-656. 2011.
Ref Type: Magazine Article

(35) Duncan PW, Wallace D, Lai SM, Johnson D, Embretson S, Laster LJ. The stroke impact scale version 2.0. Evaluation of reliability, validity, and sensitivity to change. *Stroke* 1999 October;30(10):2131-40.

(36) Duncan PW, Lai SM, Tyler D, Perera S, Reker DM, Studenski S. Evaluation of proxy responses to the Stroke Impact Scale. *Stroke* 2002 November;33(11):2593-9.

(37) Duncan PW, Bode RK, Min LS, Perera S. Rasch analysis of a new stroke-specific outcome scale: the Stroke Impact Scale. *Arch Phys Med Rehabil* 2003 July;84(7):950-63.

(38) Port van de I, Leenes K, Sellmeijer D, Zuidgeest A, Kwakkel G. Betrouwbaarheid en concurrente validiteit van de Nederlandse Stroke Impact Scale 2.0 bij patie¨nten met een CVA. *Nederlands Tijschrift voor fysiotherapie* 2008;118(1):12-8.

(39) Lamers LM, Stalmeier PFM, McDonnell J, Krabbe PFM, Busschbach JJ. Kwaliteit van leven meten in economische evaluaties: het Nederlands EQ-5D-tarief. *Nederlands Tijdschrift voor Geneeskunde* 2005;149:1574-8.

(40) Collen FM, Wade DT, Robb GF, Bradshaw CM. The Rivermead Mobility Index: a further development of the Rivermead Motor Assessment. *Int Disabil Stud* 1991 April;13(2):50-4.

(41) Hsieh CL, Hsueh IP, Mao HF. Validity and responsiveness of the rivermead mobility index in stroke patients. *Scand J Rehabil Med* 2000 September;32(3):140-2.

(42) Hsueh IP, Wang CH, Sheu CF, Hsieh CL. Comparison of psychometric properties of three mobility measures for patients with stroke. *Stroke* 2003 July;34(7):1741-5.

(43) Collin C, Wade DT, Davies S, Horne V. The Barthel ADL Index: a reliability study. *Int Disabil Stud* 1988;10(2):61-3.

(44) Collen FM, Wade DT, Bradshaw CM. Mobility after stroke: reliability of measures of impairment and disability. *Int Disabil Stud* 1990 January;12(1):6-9.

(45) Flansbjer UB, Holmback AM, Downham D, Patten C, Lexell J. Reliability of gait performance tests in men and women with hemiparesis after stroke. *J Rehabil Med* 2005 March;37(2):75-82.

(46) Nouri FM, Lincoln NB. An extended activitoes of daily living scale for stroke patients. *Clin Rehabil* 1987;1:301-5.

(47) de Haan R, Limburg M, Bossuyt P, van der MJ, Aaronson N. The clinical meaning of Rankin 'handicap' grades after stroke. *Stroke* 1995 November;26(11):2027-30.

(48) Berg K, Wood-Dauphinee S, Williams JI. The Balance Scale: reliability assessment with elderly residents and patients with an acute stroke. *Scand J Rehabil Med* 1995 March;27(1):27-36.

(49) Mao HF, Hsueh IP, Tang PF, Sheu CF, Hsieh CL. Analysis and comparison of the psychometric properties of three balance measures for stroke patients. *Stroke* 2002 April;33(4):1022-7.

(50) Sanford J, Moreland J, Swanson LR, Stratford PW, Gowland C. Reliability of the Fugl-Meyer assessment for testing motor performance in patients following stroke. *Phys Ther* 1993 July;73(7):447-54.

(51) Collin C, Wade D. Assessing motor impairment after stroke: a pilot reliability study. *J Neurol Neurosurg Psychiatry* 1990 July;53(7):576-9.

(52) Al-Janabi H, Frew E, Brouwer W, Rappange D, Van EJ. The inclusion of positive aspects of caring in the Caregiver Strain Index: tests of feasibility and validity. *Int J Nurs Stud* 2010 August;47(8):984-93.

(53) Robinson BC. Validation of a Caregiver Strain Index. *J Gerontol* 1983 May;38(3):344-8.

(54) Visser-Meily JM, Post MW, Riphagen II, Lindeman E. Measures used to assess burden among caregivers of stroke patients: a review. *Clin Rehabil* 2004 September;18(6):601-23.

(55) Brouwer WB, van Exel NJ, van GB, Redekop WK. The CarerQol instrument: a new instrument to measure care-related quality of life of informal caregivers for use in economic evaluations. *Qual Life Res* 2006 August;15(6):1005-21.

(56) Hoefman RJ, van Exel NJ, Looren de JS, Redekop WK, Brouwer WB. A new test of the construct validity of the CarerQol instrument: measuring the impact of informal care giving. *Qual Life Res* 2011 August;20(6):875-87.

(57) Bjelland I, Dahl AA, Haug TT, Neckelmann D. The validity of the Hospital Anxiety and Depression Scale. An updated literature review. *J Psychosom Res* 2002 February;52(2):69-77.

(58) Zigmond AS, Snaith RP. The hospital anxiety and depression scale. *Acta Psychiatr Scand* 1983 June;67(6):361-70.

(59) Valko PO, Bassetti CL, Bloch KE, Held U, Baumann CR. Validation of the fatigue severity scale in a Swiss cohort. *Sleep* 2008 November;31(11):1601-7.

(60) Bosscher RJ, Smit JH. Confirmatory factor analysis of the General Self-Efficacy Scale. *Behav Res Ther* 1998 March;36(3):339-43.

(61) Luszczynska A, Scholz U, Schwarzer R. The general self-efficacy scale: multicultural validation studies. *J Psychol* 2005 September;139(5):439-57.
