# Supplementary material for: Caregiver-mediated exercises with e-health support for early supported discharge after stroke (CARE4STROKE): A randomized controlled trial
Source: PLoS One. 2019 Apr 8;14(4):e0214241. doi: 10.1371/journal.pone.0214241 (PMC6453481; doi:10.1371/journal.pone.0214241)
Supplement: Supplementary file 6 [file pone.0214241.s002.docx]

6-minute walking test (total distance in meters)	159.96 (122.65) N=12	266.70 (154.66) N=20	279.43 (179.82) N=21	185.46 (83.94) N=13	259.00 (141.53) N=25	258.61 (163.83) N=28	2.49 (36.42)	P=0.946	-12.45 (21.07)
**Secondary outcome measures - Caregiver (1)**									
HADS depression (0 - 21)	2.88 (2.54)	4.85 (4.19) N=26	3.50 (3.24) N=24	4.28 (2.99)	3.58 (3.05) N=31	3.21 (2.71) N=28	-2.33 (0.77)	P=0.003	-0.72 (0.37)
HADS anxiety (0 – 21)	4.44 (3.40)	4.85 (3.89) N=26	4.63 (3.50) N=24	5.68 (2.99) N=31	5.13 (3.28) N=31	4.96 (4.08) N=28	-0.45 (0.88)	P=0.607	-0.10 (0.55)
Caregiver Strain Index (0 -13)	4.53 (2.11) N=32	5.81 (3.23) N=26	5.35 (2.95) N=23	5.42 (2.66) N=31	6.30 (3.03) N=30	5.72 (3.14) N=29	-0.54 (0.63)	P=0.393	-0.11 (0.29)
Fatigue Severity Scale mean (1 – 7)	2.54 (1.05) N=33	2.85 (1.29) N=26	2.98 (1.33) N=23	2.78 (1.61)	2.98 (1.48) N=29	2.99 (1.55) N=29	-0.21 (0.32)	P=0.499	-0.17 (0.17)
General Self-Efficacy Scale (10 – 40)	34.76 (5.04) N=33	33.42 (4.34) N=26	34.74 (4.61) N=23	34.25 (4.09)	34.29 (4.97) N=31	34.93 (3.67) N=29	1.03 (1.02)	P=0.311	0.06 (0.55)
Carer Quality of Life (0 – 14)	12.00 (1.87) N=32	5.19 (1.83) N=26	10.96 (2.16) N=23	11.69 (1.75)	5.55 (1.52) N=31	10.52 (2.03) N=29	0.62 (0.69)	P=0.368	0.14 (0.28)
